# Characteristics of paced waveforms and current of injury prior to septal perforation during left bundle branch area pacing

**DOI:** 10.1093/europace/euag185

**Published:** 2026-07-20

**Authors:** Maciej Dyrbuś, Samuel Stempfel, Valérian Valiton, Heli Tolppanen, Haran Burri

**Affiliations:** Cardiac Pacing Unit, Department of Cardiology, University Hospital of Geneva, rue Gabrielle Perret Gentil 4, Geneva 1211, Switzerland; 3rd Department of Cardiology, School of Medical Sciences in Zabrze, Silesian Center for Heart Diseases, Medical University of Silesia, Katowice, Poland; Cardiac Pacing Unit, Department of Cardiology, University Hospital of Geneva, rue Gabrielle Perret Gentil 4, Geneva 1211, Switzerland; Cardiac Pacing Unit, Department of Cardiology, University Hospital of Geneva, rue Gabrielle Perret Gentil 4, Geneva 1211, Switzerland; Cardiac Pacing Unit, Department of Cardiology, University Hospital of Geneva, rue Gabrielle Perret Gentil 4, Geneva 1211, Switzerland; Heart and Lung Center, Helsinki University Central Hospital, Helsinki, Finland; Cardiac Pacing Unit, Department of Cardiology, University Hospital of Geneva, rue Gabrielle Perret Gentil 4, Geneva 1211, Switzerland

**Keywords:** Left bundle branch pacing, Perforation, Intracardiac electrogram, Current of injury

Left bundle branch area pacing (LBBAP) is being increasingly adopted.^1–3^ Implantation is associated with a non-negligible risk of septal perforation.^4–6^ Current of injury (COI) derived from the unfiltered unipolar intracardiac electrogram (iEGM) channel has been proposed as a marker of lead depth.^5,7,8^ Previous studies have focused predominantly on sensed COI, demonstrating that low amplitudes may indicate perforation.^7,9^ With increasing use of continuous pacing during lead advancement, paced COI is of particular interest. We aimed to characterize morphological changes in paced iEGM waveforms which may herald, and thereby avoid, perforation.

Consecutive patients included in the Geneva Conduction System Pacing registry (approved by the local ethics committee) were included. Microperforation involves protrusion of the screw into the left ventricular cavity with preserved capture, which is difficult to diagnose without concomitant imaging, whereas macroperforation is clear with loss of unipolar capture at 5 V/0.5 ms output.^7^ We therefore restricted analysis to patients who had macroperforation during continuous pacing. If >1 perforation occurred in a patient, only the first was included.

Leads were deployed during continuous unipolar pacing at 5 V/0.5 ms via the rotational John-Jiang connector cable or clips fixed to the lead stylet. Unipolar iEGMs were recorded using the Boston Scientific Labsystem Pro system with 0.5–500 Hz filters. Measurements were performed using digital callipers.

Paced COI and Q-wave amplitude/duration were analysed during their deployment and at the last captured cycle before macroperforation. The paced waveforms at final lead position were analysed for comparison. The number of paced beats and time between the first appearance of predefined events (paced COI <10 mV, paced COI <5 mV, or appearance of a paced Q-wave) and macroperforation was evaluated. All measurements were performed by two authors (M.D. and S.S). All uncertainties regarding measurement boundaries were resolved by consensus discussion with the corresponding author. Intra- and inter-observer reproducibility of measurements were tested in 30 randomly selected waveforms using the Bland–Altman analysis.

A total of 67/925 (7.2%) patients implanted with LBBAP had macroperforation. Of these, 21 patients (age 83.5 ± 3.8 years, 12 males, left ventricular ejection fraction 54%±8%, interventricular septal thickness 9.5 ± 1.3 mm, 7 ischaemic heart disease) had perforation during continuous pacing and were included. The remaining 46 patients with macroperforation were excluded because the events did not occur during continuous pacing (e.g. while deploying a disconnected lumenless lead before the John Jiang rotational connector was available at our centre), thereby not allowing iEGM analysis of all paced cycles directly preceding perforation. Leads were stylet-driven in 15 patients and lumenless in 6 patients. The results are shown in Figure 1. Data are shown as median [interquartile range]. The intra- and inter-observer reproducibility (bias and 95% limits of agreement) were 0.00 (−0.22 to 0.22) mV and −0.02 (−0.26 to 0.22) mV, respectively. Paced COI at septal entry was 9.0 [6.3–11.7] mV, increasing to a maximum of 26.5 [20.3–28.3] mV during lead advancement. In all patients, the paced COI fell to <10 mV, at 22 [11–69] beats (16 [6–52] s) before perforation. In 15 (71.4%) patients, the COI fell to <5 mV at 9 [1–24] beats (6 [1–18] s) before perforation, whereas 6 (28.6%) patients had COI >5 mV at their last captured beat before perforation. At the final position, the paced COI amplitude was 13.4 [10.2–17.2] mV.

**Figure 1:**
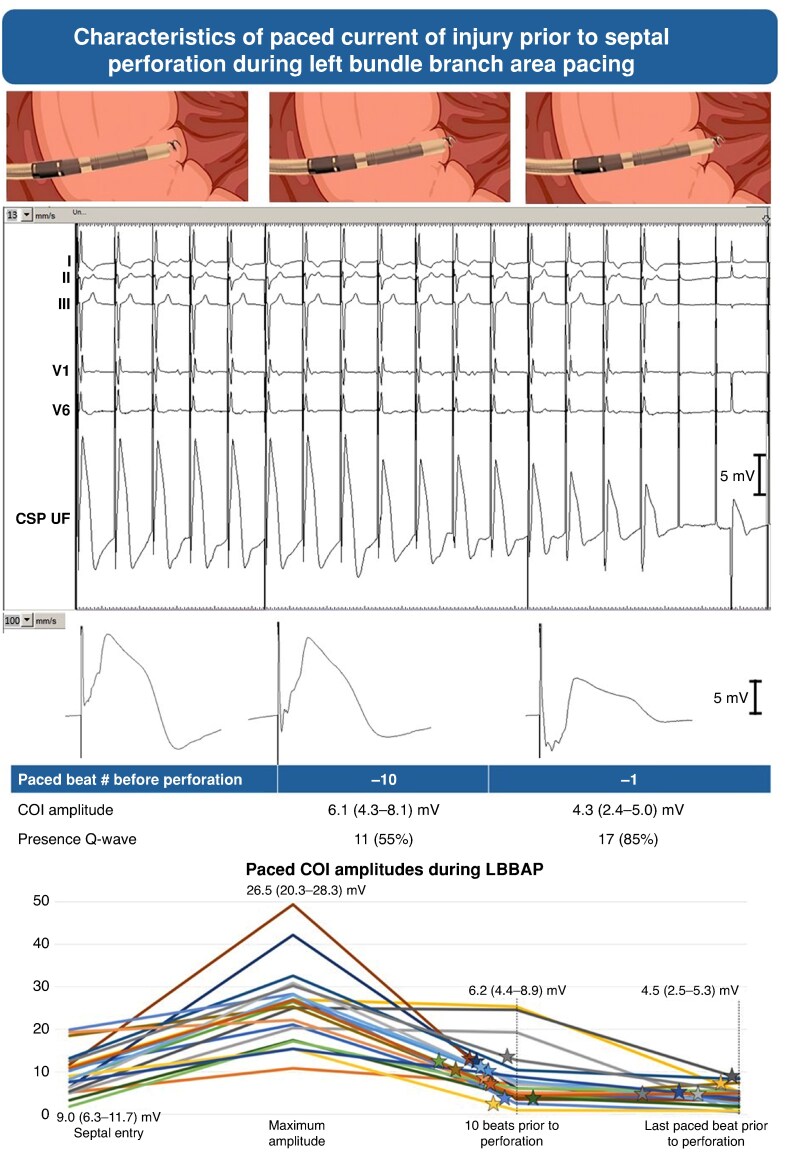
Paced intra-cardiac waveforms with progression of the lead from the left subendocardium to microperforation and macroperforation (illustrated on the top of the panel). Note the fall in paced COI amplitude, which approaches, but does not drop to <5 mV, with appearance of a deep and broad Q-wave before macroperforation and loss of capture (last two spikes) followed by a sensed beat (with a deep Q-wave and COI amplitude <5 mV). In the bottom panel, the paced COI amplitudes during LBBAP implantation leading to macroperforation in all analysed patients can be appreciated, at four timepoints: septal entry, maximum amplitude obtained during lead advancement, paced COI amplitudes at 10 beats prior to perforation, and the last paced beat before perforation. The stars, coloured according to the corresponding line, indicate the amplitude of paced COI when the first Q wave occurred. Values are presented as median (Quartile 1–Quartile 3). COI, current of injury; CSP-UF, conduction system pacing unfiltered channel (0.5–500 Hz); LBBAP, left bundle branch area pacing.

A paced Q-wave was observed in 18 (85.7%) patients, appearing at 15 [7–45] beats (14 [4–38] seconds) before perforation, with 5.0 [2.9–8.5] mV amplitude and 68 [60–98] ms duration at the last captured beat. In 7 patients, the Q-wave appeared with COI>10 mV. At final lead position, a paced Q-wave was observed in 5 (23.8%) patients, with an amplitude of 4.2 [3.0–4.6] mV and duration of 50 [46–59] ms (similar to during perforation).

Our preliminary findings indicate that a drop in COI to <10 mV and/or appearance of a Q-wave can forewarn septal perforation during LBBAP implantation. Therefore, if either of these signs appears, it is wise to check pacing impedance and transiently interrupt pacing to evaluate the sensed iEGM waveform, especially as sensed COI amplitude may differ compared to during pacing.^10^ The presence of a fascicular potential is an additional sign indicating that the left septal subendocardium has been reached. Signs of micro-perforation on the *sensed* iEGM have been described elsewhere, and include deep and wide Q or S-wave, and Q or S amplitude > COI amplitude, which however depends on wavefront activation and is only true in patients who are in intrinsic rhythm with a narrow QRS or non-left bundle branch block.^7^ During pacing, the wavefront has the advantage of being standardized with centrifugal activation in all cases. Another criterion for perforation is *sensed* COI <5 mV.^9^ Our findings suggest that *paced* COI <5 mV may not be able to forewarn macroperforation, as in 28.6% of our cases, the lead perforated before falling to <5 mV, presumably due to rapid progression in the septum.^7^

It is important to note that a paced Q-wave was observed in a subset of patients with optimal final lead position, however with a high COI amplitude.

This was a single-centre, retrospective analysis with a relatively small number of cases. Signal acquisition was performed using a single system and filter setting, which may limit applicability to other platforms and pacing system analysers. The rate of lead progression in the septum is affected by the substrate, lead type, rapidity of rotations, and applied forward force. These factors differed between patients and were likely to have impacted the number of paced beats before perforation.

Paced iEGM waveforms should be carefully monitored during LBBAP lead deployment, as they provide valuable information which may herald, and thus avoid, septal perforation.

## Data Availability

The data underlying this article will be shared on reasonable request to the corresponding author.
